# MicroRNA-33b is a Potential Non-Invasive Biomarker for Response to Atorvastatin Treatment in Chilean Subjects With Hypercholesterolemia: A Pilot Study

**DOI:** 10.3389/fphar.2021.674252

**Published:** 2021-05-21

**Authors:** Carmen Gloria Ubilla, Yalena Prado, Jeremy Angulo, Ignacio Obreque, Isis Paez, Nicolás Saavedra, Kathleen Saavedra, Tomás Zambrano, Luis A. Salazar

**Affiliations:** ^1^ Center of Molecular Biology and Pharmacogenetics, Department of Basic Sciences, Faculty of Medicine, Universidad de La Frontera, Temuco, Chile; ^2^ Department of Medical Technology, Faculty of Medicine, Universidad de Chile, Santiago, Chile

**Keywords:** hypercholesterolemia, circulating microRNAs, atorvastatin, epidrugs, statins

## Abstract

Evidence accumulated so far indicates that circulating levels of microRNAs (miRNAs) are associated with several pathologies. Therefore, differential expression of extracellular miRNAs exhibits promising potential for screening and diagnosis purposes. We evaluated plasma miRNAs in response to the lipid-lowering drug atorvastatin in patients with hypercholesterolemia (HC) and controls. Methods: We selected miRNAs based on previous data reported by our group and also by employing bioinformatics tools to identify 10 miRNAs related to cholesterol metabolism and statin response genes. Following miRNA identification, we determined plasma levels of miRNA-17-5p, miRNA-30c-5p, miRNA-24-3p, miRNA-33a-5p, miRNA-33b-5p, miRNA-29a-3p, miRNA-29b-3p, miRNA-454-3p, miRNA-590-3p and miRNA-27a-3p in 20 HC patients before and after 1 month of 20 mg/day atorvastatin treatment, evaluating the same miRNA set in a group of 20 healthy subjects, and employing qRT-PCR to determine differential miRNAs expression. Results: HC individuals showed significant overexpression of miRNA-30c-5p and miRNA-29b-3p vs. NL (*p* = 0.0008 and *p* = 0.0001, respectively). Once cholesterol-lowering treatment was concluded, HC individuals showed a substantial increase of three extracellular miRNAs (miRNA-24-3p, miRNA-590, and miRNA-33b-5p), the latter elevated more than 37-fold (*p* = 0.0082). Conclusion: Data suggest that circulating miRNA-30c-5p and miRNA-29b-3p are associated with hypercholesterolemia. Also, atorvastatin induces a strong elevation of miRNA-33b-5p levels in HC individuals, which could indicate an important function that this miRNA may exert upon atorvastatin therapy. Additional studies are needed to clarify the role of this particular miRNA in statin treatment.

## Introduction

Ever since the discovery that miRNAs can circulate in the extracellular medium with extraordinary stability, there has been substantial interest in identifying these molecules as non-invasive biomarkers for several problematic pathologies to public health, such as cardiovascular disease. Advancements made so far show that miRNAs are not only expressed differently between a varied number of conditions, but their ability to be measured in diverse bodily fluids such as serum or plasma, urine, tears, breast milk, amniotic fluid, cerebrospinal fluid, saliva, and semen show promise for screening, diagnosis, prognosis, and follow-up purposes.

It is well-established that abnormal lipid accumulation sets an atherogenic milieu (Summerhill and et al., 2019), and to date, numerous miRNAs have been reported to be involved in lipid metabolism (Fernández-Hernando et al., 2011; Aryal et al., 2017). We previously showed that miRNAs expression is altered *in vitro* (Zambrano et al., 2015) and *in vivo* (Zambrano et al., 2018) following statin treatment, a lipid-lowering drug of widespread use that also reduces the cardiovascular risk (Garcia-Gil et al., 2018). However, the improvement in lipid profiles is usually underachieved, as almost half of the patients undergoing statins will show a suboptimal response to these drugs (Gitt et al., 2012; Akyea et al., 2019). Moreover, very little is known about the potential use of miRNAs as biomarkers of poor response to statin therapy, which would ultimately help detect patients not meeting appropriate LDL-C reduction goals. Therefore, we carried out bioinformatics and relative expression studies to determine circulating miRNAs in healthy subjects and patients with hypercholesterolemia undergoing treatment with 20 mg/day atorvastatin for one month, to identify candidate miRNAs as potential therapeutic targets.

## Materials and Methods

### Subjects

A total of forty individuals were selected for this study. Twenty subjects were normolipidemic and comprised the control group, whereas 20 were hypercholesterolemic patients treated with 20 mg/day atorvastatin for 1 month. Biochemical profiles were performed on all patients, and the treated group was sampled before and after completion of the lipid-lowering treatment. The patients were recruited from public health centers. Blood samples were then processed to separate the plasma, which was frozen for further analysis. The Ethics Committee of University of La Frontera (Protocol #045_17) approved the study protocol. All subjects gave their written informed consent to participate from this investigation.

### Biochemical Analyses

To establish plasma lipid levels before and after atorvastatin treatment, blood collection was performed by direct venous puncture following an overnight fast using EDTA tubes. Total cholesterol, HDL cholesterol (HDL-C) and triglycerides (TG) were measured by routine enzymatic-colorimetric methods. LDL cholesterol (LDL-C) was calculated using the Friedewald equation when TG levels were not above 400 mg/dl.

### Selection of miRNAs

As aforementioned, we based the current selection of ten circulating miRNAs on previous data obtained by our group (Zambrano et al., 2015; Zambrano et al., 2018). Briefly, in the first report, we identified deregulated miRNAs by analyzing their expression profile in HepG2 cells treated during 24 h with different statins. In the second study, we employed multiple bioinformatic tools such as TargetScan, miRanda, DianaLab, MicroCosm, and PicTar to narrow down the previous *in vitro* miRNA data, evaluating a subset of 84 miRNAs potentially associated with 28 key genes involved in cholesterol metabolism and statin response in subjects undergoing 1 month of different low-dose statins. In the present study, we crossed our previous results and updated the selection criteria by running Pharmaco miR, a miRNA pharmacogenomics database that identifies associations of miRNAs and both the genes they regulate and the drugs annotated, to finally select the ten most probably dysregulated miRNAs in patients with hypercholesterolemia following a 20 mg/day atorvastatin dose for 4 weeks and controls.

### RNA Extraction

Circulating miRNAs were extracted using the miRNeasy serum/plasma kit (Quiagen, Hilden, Germany) according to the manufacturer’s instruction, using 200 µl of plasma. miR-39 of *C. elegans* was incorporated as a spike-in control to normalize quantification (1.6 × 10^8^ copies/µl). RNA concentration and purity were determined by spectrophotometry (Infinite^®^ 200 PRO NanoQuant) using the 260 nm/280 nm absorbance ratio. The samples were diluted to a concentration of 5 ng/μL and stored at −80°C for ulterior use.

### Reverse Transcription

The cDNA synthesis was performed using the TaqMan^™^ Advanced miRNA cDNA synthesis kit. The protocol carried out consisted of four steps: 1) polyadenylation, 2) ligation of adapters, 3) retrotransciption and 4) miRNAs obtention. Afterward, miRNAs obtained were stored at −20°C for later use.

### qPCR Expression Analyses

Each sample was diluted to 1:10 with ultrapure water. The PCR Mix was prepared in sufficient quantity for each assay, for the identification of miRNA-17-5p, miRNA-30c-5p, miRNA-24p-3p, miRNA-33a-5p, miRNA-33b-5p, miRNA-29a-3p, miRNA-29b-3p, miRNA-454-3p, miRNA-590-3p and miRNA-27-3p following the manufacturer’s protocol.

### Interaction Network of Differentially Expressed miRNAs

To generate the interaction network of selected miRNAs, we employed miRTargetLink 2.0 (Kern and et al., 2021), a tool containing experimentally validated interactions on human microRNA-mRNA pairs. Data shown correspond to miRNA-target interactions with strong support, i.e., validated experimentally by reporter assay, western blot, qPCR, microarray, and/or next-generation sequencing experiments. The software obtains miRNAs annotations from the latest version of miRBase (v.22.1), while the experimentally validated targets are retrieved from miRTarBase (v.8) (Huang et al., 2020) and miRATBase (Kern et al., 2021). miRTargetLink 2.0 can be freely accessible from the following link (https://ccb-compute.cs.uni-saarland.de/mirtargetlink2/).

### Statistical Analysis

Data were analyzed using GraphPad Prism version 5.00 for Windows (GraphPad Software, San Diego, CA, United States). Normal distribution was assessed using D’Agostino-Pearson testing. As data came from a limited set of ten miRNAs, correction for multiple testing was not employed, and differential miRNA expression was calculated using 2^−ΔCT^ to compare normolipidemic and hypercholesterolemic subjects. The Mann-Whitney test was applied for unpaired data and the Wilcoxon test for paired data. A *p*-value <0.05 was considered statistically significant.

## Results

### Clinical Data, Plasma Lipids and Statin Response

Table 1 shows anthropometric data and lipid profiles of normolipidemic (NL) and hypercholesterolemic (HC) patients before and after 20 mg/day of atorvastatin during a 4-weeks period. Significant differences were observed for all lipid levels except for HDL-C.

**TABLE 1:** Clinical, demographics and plasma lipid levels before and after treatment with atorvastatin (20 mg/day/4 weeks) of normolipidemic (NL) and hypercholesterolemic (HC) individuals.

Parameter	NL	HC	HC post-treatment	% Change	*p*-value
Female/Male	15/5	15/5	—	—	-—
Age (years)	31.2 ± 7.3	47.3 ± 11.3	—	—	—
BMI (kg/m^2^)	26.4 ± 4.4	27.0 ± 3.0	—	—	—
Total cholesterol (mg/dl)	151.3 ± 28.4	239.3 ± 28.2	158.1 ± 33.4	34.1 ± 10.7	<0.001
LDL-cholesterol (mg/dl)	87.5 ± 17.4	176.0 ± 15.5	96.1 ± 29.9	44.6 ± 14.0	<0.001
HDL-cholesterol (mg/dl)	50.6 ± 19.7	44.4 ± 10.0	41.2 ± 9.4	6.4 ± 14.9	0.056
Triglycerides (mg/dl)	80.2 ± 30.5	150.4 ± 66.2	121.6 ± 55.4	10.9 ± 37.8	<0.05
VLDL-cholesterol (mg/dl)	17.0 ± 6.5	27.0 ± 7.0	21.6 ± 6.2	15.0 ± 36.7	<0.05

Values expressed as mean ± standard deviation, n, number of subjects; BMI: body mass index, LDL-C: low-density lipoprotein cholesterol, HDL-C: High-density lipoprotein cholesterol, VLDL: Very low-density lipoprotein cholesterol.

### Expression of Circulating miRNAs Associated With Cholesterol Metabolism and Response to Statins

**FIGURE 1:**
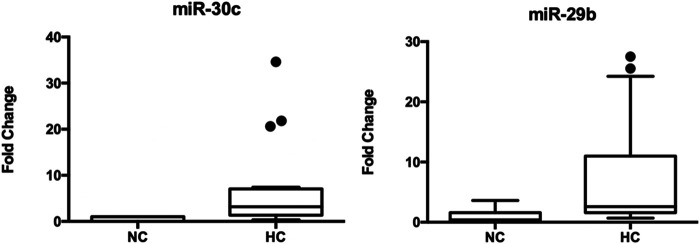
Expression of circulating miRNA-30c and miRNA-29b in hypercholesterolemic patients vs. controls. *Mann-Whitney test. *C. elegans* miRNA-39 was used as a normalizing control. The values of the threshold cycle (Ct) of each miRNA, normalized against this sequence (ΔCt = Ct target miRNA—Ct endogenous control), the plasma expression levels of these miRNAs were calculated according to 2^−ΔCt^ method. The total number of samples analyzed was 20 for each study group.

We found two extracellular miRNAs differentially expressed between normolipidemic (NL) and hypercholesterolemic (HC) individuals. Figure 1 shows the fold change of miRNA-30c and miRNA-29b in these subjects. The rest of the miRNAs evaluated did not show significant differences between the study groups.

### Expression of Circulating miRNAs in Hypercholesterolemic (HC) Patients and Response to Atorvastatin Treatment

According to the expression of extracellular miRNAs in patients at the beginning and post-treatment, significant differences were observed for miRNA-33-5p, miRNA-24-3p and miRNA-590. Table 2 illustrates the relative expression of miRNAs before and after 1 month with atorvastatin (20 mg/day).

**TABLE 2:** Circulating miRNA levels in hypercholesterolemic (HC) subjects before and following 20 mg/day/4 weeks atorvastatin treatment.

miRNA	HC basal	HC post-treatment	p-value*
miR-17-5p	1 ± 1.583	4.727 ± 8.082	0.1297
miR-30c	1 ± 1.314	0.789 ± 1.140	0.7983
miR-33a-5p	1 ± 1.408	4.230 ± 9.728	0.2413
miR-33b-5p	1 ± 1.644	37.479 ± 111.932	0.0082
miR-29a	1 ± 1.466	3.057 ± 7.289	0.4900
miR-29b	1 ± 1.189	3.031 ± 4.002	0.0799
miR-454	1 ± 1.389	2.750 ± 3.968	0.2253
miR-24-3p	1 ± 1.271	4.815 ± 6.527	0.0494
miR-590	1 ± 1.791	9.207 ± 12.656	0.0047
miR-27a-3p	1 ± 2.239	2.161 ± 3.597	0.1336

* Wilcoxon matched-pairs signed rank test. The plasma expression levels of these miRNAs were calculated according to 2 - ^ΔΔCt^ [ΔΔCt = Ct Basal (target miRNA—Ct endogenous control)—Ct treatment (target miRNA—Ct endogenous control)].

### Correlation of miRNAs and Lipid Reduction

Table 3 shows the correlation between basal circulating miRNAs and the reduction percentage (%) of LDL-C following 1 month of treatment with a 20 mg/day atorvastatin dose. All miRNAs showed positive correlation with LDL-C reductions, but only five (miRNA-30c, miRNA-29a, miRNA-454, miRNA-24-3p and miRNA-590) were statistically significant. Supplementary Figure S1 displays the correlation of circulating miRNAs and LDL-C reduction.

**TABLE 3:** Correlation analysis of miRNAs and the percentage of lipid reduction after statin therapy.

miRNA	Slope	*R* ^2^	*p*-value
miRNA-17-5p	0.393 ± 0.336	0.0703	0.2583
miRNA-30c	0.495 ± 0.172	0.313	0.0103*
miRNA-33a-5p	2.845 ± 1.484	0.169	0.0712
miRNA-33b-5p	9.434 ± 6.099	0.117	0.1393
miRNA-29a	1.765 ± 0.759	0.230	0.0321*
miRNA-29b	0.666 ± 0.786	0.038	0.4080
miRNA-454	1.763 ± 0.807	0.209	0.0425*
miRNA-24-3p	2.318 ± 1.047	0.205	0.0393*
miRNA-590	16.97 ± 7.239	0.244	0.0314*
miRNA-27a-3p	0.084 ± 0.075	0.065	0.2753

* Statistically significant.

### Predicted Interactions of Deregulated miRNAs

**FIGURE 2:**
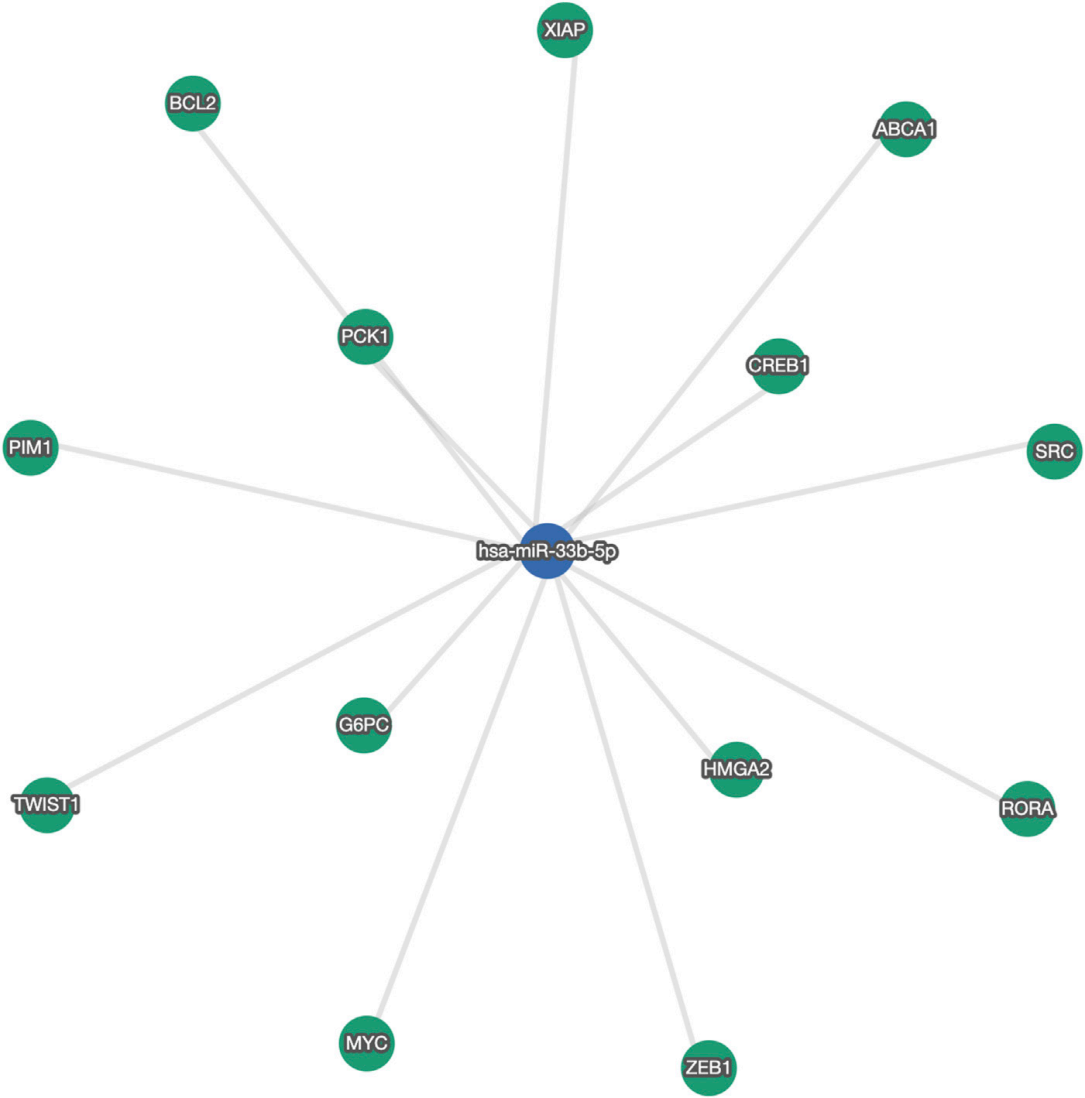
Network of validated gene targets for miRNA-33b-5p. ***ABCA1***: ATP binding cassette subfamily A member one; ***BCL2***: B-cell lymphoma two Apoptosis Regulator; ***CREB1***: cAMP responsive element binding protein one; ***G6PC***: glucose-6-phosphatase, catalytic; ***HMGA2***: High Mobility Group AT-Hook two; ***MYC***: MYC Proto-Oncogene, BHLH Transcription Factor; ***PCK1***: Phosphoenolpyruvate carboxykinase one; ***PIM1***: Proto-Oncogene, Serine/Threonine Kinase; ***RORA***: RAR Related Orphan Receptor A; ***SRC***: SRC Proto-Oncogene, Non-Receptor Tyrosine Kinase; ***TWIST1***: Twist Family BHLH Transcription Factor 1; ***XIAP***: X-linked inhibitor of apoptosis; ***ZEB1***: zinc finger E-box binding homeobox 1.

We found five deregulated miRNAs, two of them were overexpressed in hypercholesterolemic vs. normal subjects (miRNA-30c-5p and miRNA-29b-3p), while three were significantly upregulated in HC patients after concluding atorvastatin treatment (miRNA-24-3p, miRNA-590, and miRNA-33b-5p). Importantly, miRNA-33b-5p showed a substantial 37-fold increase since treatment initiation. Figure 2 shows a network of validated mRNA targets for miRNA-33b-5p. Additional miRNA-mRNA pairs interaction networks from the remaining deregulated miRNAs are presented as supplementary material (Supplementary Figures S2–S5).

## Discussion

The LDL-C lowering efficacy for a 20 mg atorvastatin dose is reported to be around 42% (Adams et al., 2015). In our study, half of the patients did not achieve this goal, and a quarter showed LDL-C reductions lower than 27%. This highly heterogeneous response to statins is well documented (Karlson et al., 2016) and is consistent with previous reports made by our group (Rosales et al., 2012). As for miRNAs, we observed an up-regulation of two miRNAs, i.e., miRNA-30c and miRNA-29b, between patients having abnormally high cholesterol levels vs. healthy subjects. Previously, Soh and colleagues reported that miRNA-30c reduce lipid synthesis and LDL-precursors due to its interaction with the 3-UTR of the microsomal triglyceride transfer protein (MTP) (Soh et al., 2013), a protein central for lipoprotein assembly and production of LDL precursors. Hence, miRNA-30c contributes to control hepatic and plasma lipids by decreasing hyperlipidemia, which is a likely explanation of why this miRNA is elevated in hypercholesterolemic individuals. Moreover, Sodi et al. reported a significant and positive correlation between miRNA-30c with total cholesterol (TC) and LDL-C (Sodi et al., 2017), which further demonstrates an important regulatory role for this miRNA in lipid homeostasis. Authors also showed that pravastatin but not rosuvastatin increased serum miRNA-30c, which points toward a dissimilar effect of statins on miRNAs expression, even though both statins assessed share a hydrophilic nature. One reason supporting this differential regulation could be the differing treatment periods (1-year pravastatin vs. 8-weeks rosuvastatin). Likewise, we have previously reported that different statins produce variable effects on miRNA expression (Zambrano et al., 2015; Zambrano et al., 2018). This distinctive effect has also been documented at different levels of gene regulation (Leszczynska et al., 2011). As far as we know, this is the first study disclosing the relationship between miRNA-30c and atorvastatin treatment.

The most considerable finding observed is a 37-fold increase of miRNA-33b, together with a significant elevation of circulating miRNA-590 and miRNA-24-3p levels in HC patients upon completion of atorvastatin treatment. The miRNA-33b is part of the miRNA-33 family, formed by miRNAs 33a and 33b. This pair represent one of the most widely characterized miRNA mediators involved in lipid homeostasis. Both are intronic miRNAs differing by 2-nucleotides, but unlike miRNA-33a, miRNA-33b is encoded within the sterol regulatory element-binding protein-1 (*SREBP1*), a transcription factor implicated in fatty acid metabolism. Existing data connecting miRNA-33b and statins are abundant. Reports show that miRNA-33b is co-transcribed along *SREBP1*, working coordinately to control lipid levels (Marquart et al., 2010; Najafi-Shoushtari et al., 2010; Rayner et al., 2010; Davalos et al., 2011). Regarding statins, investigations display mixt outcomes. In 2012, Takwi et al. showed an upregulation of miRNA-33b expression following lovastatin treatment of medulloblastoma cells (Takwi et al., 2012). 4 years later, Zhang and colleagues demonstrated that pitavastatin reverted the oxLDL-mediated suppression of miRNA-33b in human THP-1 cells (Zhang et al., 2016). Additional evidence supporting a relation between statins and miRNA-33 came from studying statin-naïve subjects with metabolic syndrome (MetS) (Chen et al., 2016). The authors showed that MetS subjects had significantly higher plasma values of miRNA-33 than their healthy controls counterparts. Afterward, MetS subjects treated with atorvastatin or pitavastatin experimented an additional increase in circulating miRNA-33 levels. Moreover, when studying the murine macrophage cell line RAW264.7 and bone marrow-derived macrophages (BMDM), authors found a dose-dependent up-regulation of miRNA-33 in both cell lysates and medium, regardless of the statin used. Our previous assessment of statin treatment on miRNAs from hepatocellular carcinoma cells (HepG2) revealed that neither miRNA-33a nor miRNA-33b was affected by low-dose atorvastatin or simvastatin (Zambrano et al., 2015). However, in peripheral blood mononuclear cells (PBMC) (Zambrano et al., 2018), we observed that atorvastatin but not simvastatin repressed the cellular miRNA-33b content in humans, representing a contrasting outcome from the MetS cohort. Nonetheless, several differences must be underlined, especially regarding the subjects investigated. First, in the study from (Chen et al., 2016), included individuals were defined by traditional MetS criteria, none of which considers high LDL-C levels, while we specifically included individuals having elevated LDL-C concentrations according to clinical characteristics from both studies (MetS: 155 mg/dl; PBMC: 182 mg/dl). Second, our cohort was treated during 1 month with a 10 mg/day statin dose after a thirty-day wash-out period, vs. the 3-months statin therapy for statin-naïve MetS individuals. In addition to the dissimilar ethnic background between Taiwanese and Brazilians, and the fact that we did not evaluate circulating miRNAs, another critical difference is represented by the cellular model employed. While we studied a variety of peripheral cells containing a single, rounded nucleus such as lymphocytes and monocytes i.e., PBMC, (Chen et al., 2016) specifically assessed macrophages. Even though macrophages are differentiated from monocytes, macrophages represent a very specialized and highly heterogeneous cellular type with a distinct phenotype and functionality than monocytes due to their particularly active role as one of the first lines of immune defense. Lastly, our study evaluated the miRNA-33 family separately, i.e., miRNA-33a and miRNA-33b, in contrast to what was displayed by (Chen et al., 2016), where they showed that the upregulation induced by atorvastatin affected miRNA-33 generally, making its specific impact on miRNA-33a or 33-b indistinguishable from one another.

On the other hand, the evidence surrounding the role that miRNA-590 and miRNA-24-3p portray in statin therapy is scarce. Studies confer miRNA-590 a function in lipid homeostasis by inhibiting lipoprotein lipase (LPL), an enzyme that degrades circulating triglycerides, resulting in attenuated lipid accumulation in human THP-1 macrophages (He et al., 2014). Regarding statins, we previously showed that Brazilian patients not meeting their expected LDL-C reduction goal following 10 mg/day atorvastatin had a significant downregulation of this particular miRNA in PBMC (Zambrano et al., 2018). Conversely, the present study’s data revealed a >9-fold increase in circulating levels of miRNA-590 following 20 mg/day atorvastatin. This conflicting miRNA behavior is not clear, but one key difference is that presently, we did not assess miRNA performance according to subgroups of LDL-C goals achievements. Besides, we evaluated extracellular rather than intracellular miRNAs. In the case of miRNA-24-3p, studies show that its expression is significantly increased in the livers of high-fat diet-treated mice (Ng et al., 2014). The same study revealed insulin-induced gene 1 (*Insig1*) -a lipogenesis inhibitor-as a validated target of miRNA-24. Therefore, elevated miRNA-24 levels decrease hepatic lipid accumulation *via Insig1* up-regulation. In the same way, Wang et al. (Wang et al., 2018) showed that obesity-induced miRNA-24 overexpression inhibited Scavenger Receptor B1 (*SR-B1*), a member of the CD36 family of scavenger receptors B that facilitates selective cholesterol uptake from high-density lipoproteins (HDL). Additionally, miRNA-24 increased the expression of important genes related to cholesterol synthesis, such as 3-hydroxy-3-methyl-glutaryl-coenzyme A reductase (*HMGCR*) and sterol regulatory element-binding protein 2 (*SREBF2*). Therefore, miRNA-24 assists in regulating cholesterol homeostasis and steroidogenesis by repressing HDL uptake in HepG2 cells. However, its role regarding statin therapy remains to be clarified.

One of the main limitations of the present study is its small sample size, preventing us from reporting more robust conclusions. Even though it did not restrict the successful identification of extracellular miRNA deregulation, our preliminary data should be interpreted in light of the limited cohort evaluated. We emphasize the need for additional in-depth stratified analysis on larger population and functional studies in different models to clarify further the relationship between statins and miRNAs, as they represent promising candidates for therapeutic manipulation.

## Data Availability

The raw data supporting the conclusions of this article will be made available by the authors, without undue reservation, to any qualified researcher.
