# Supplementary material for: MicroRNA-33b is a Potential Non-Invasive Biomarker for Response to Atorvastatin Treatment in Chilean Subjects With Hypercholesterolemia: A Pilot Study
Source: Front Pharmacol. 2021 May 21;12:674252. doi: 10.3389/fphar.2021.674252 (PMC8175777; doi:10.3389/fphar.2021.674252)
Supplement: Supplementary file 1 [file DataSheet1.docx]

**Supplementary Material**

1. **Correlation analysis of miRNAs-lipid reduction**

**Figure S1.** Correlation analysis of 10 miRNAs and lipid reductions percentages following a 20 mg/day atorvastatin dose treatment in hypercholesterolemic patients.

1. **Interaction network of deregulated miRNAs.**


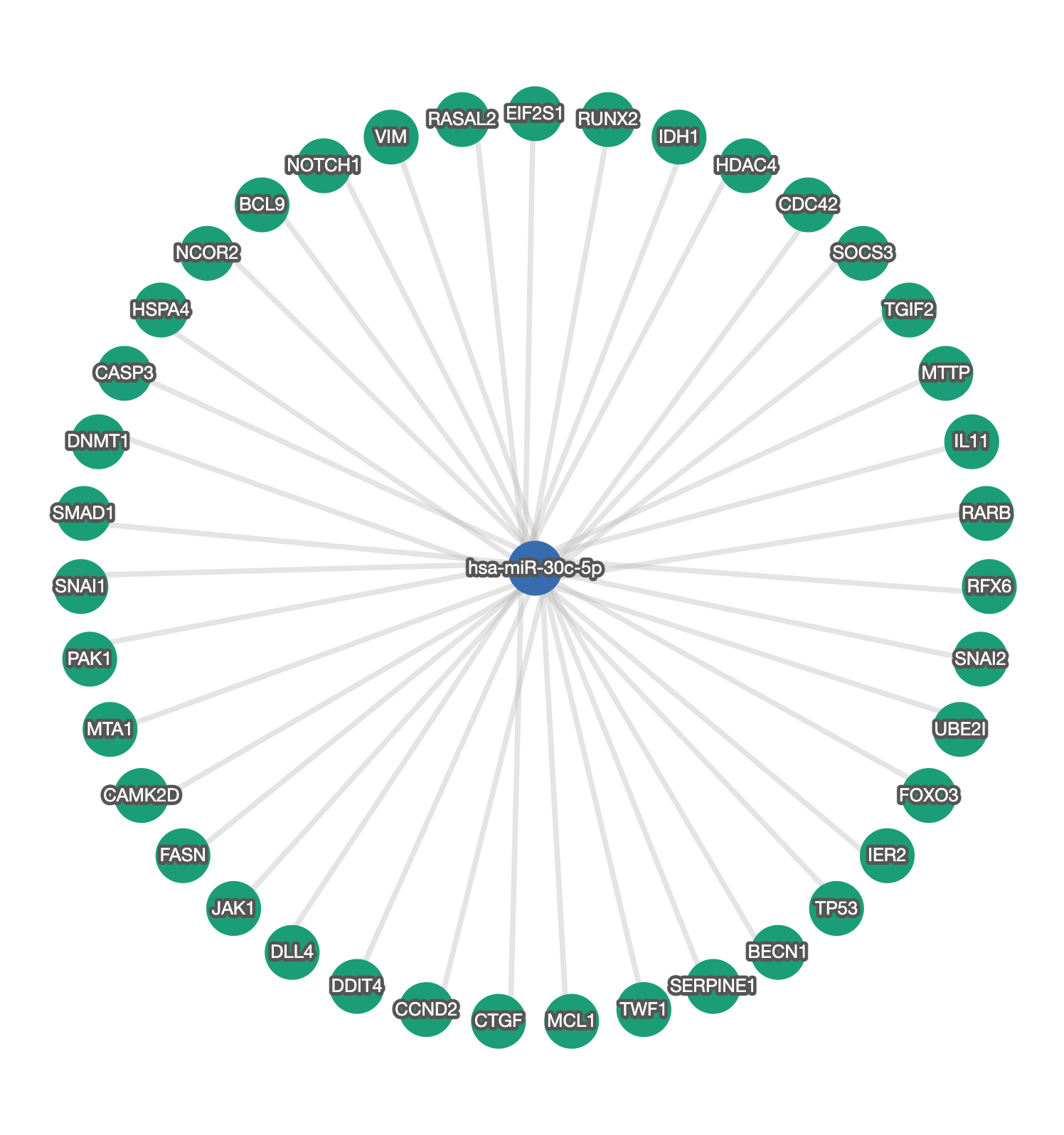


**Figure S2.** Interaction network of miRNA-30c-5p and its validated target genes. Data was retrieved using miRTargetLink 2.0. (https://ccb-compute.cs.uni-saarland.de/mirtargetlink2/).


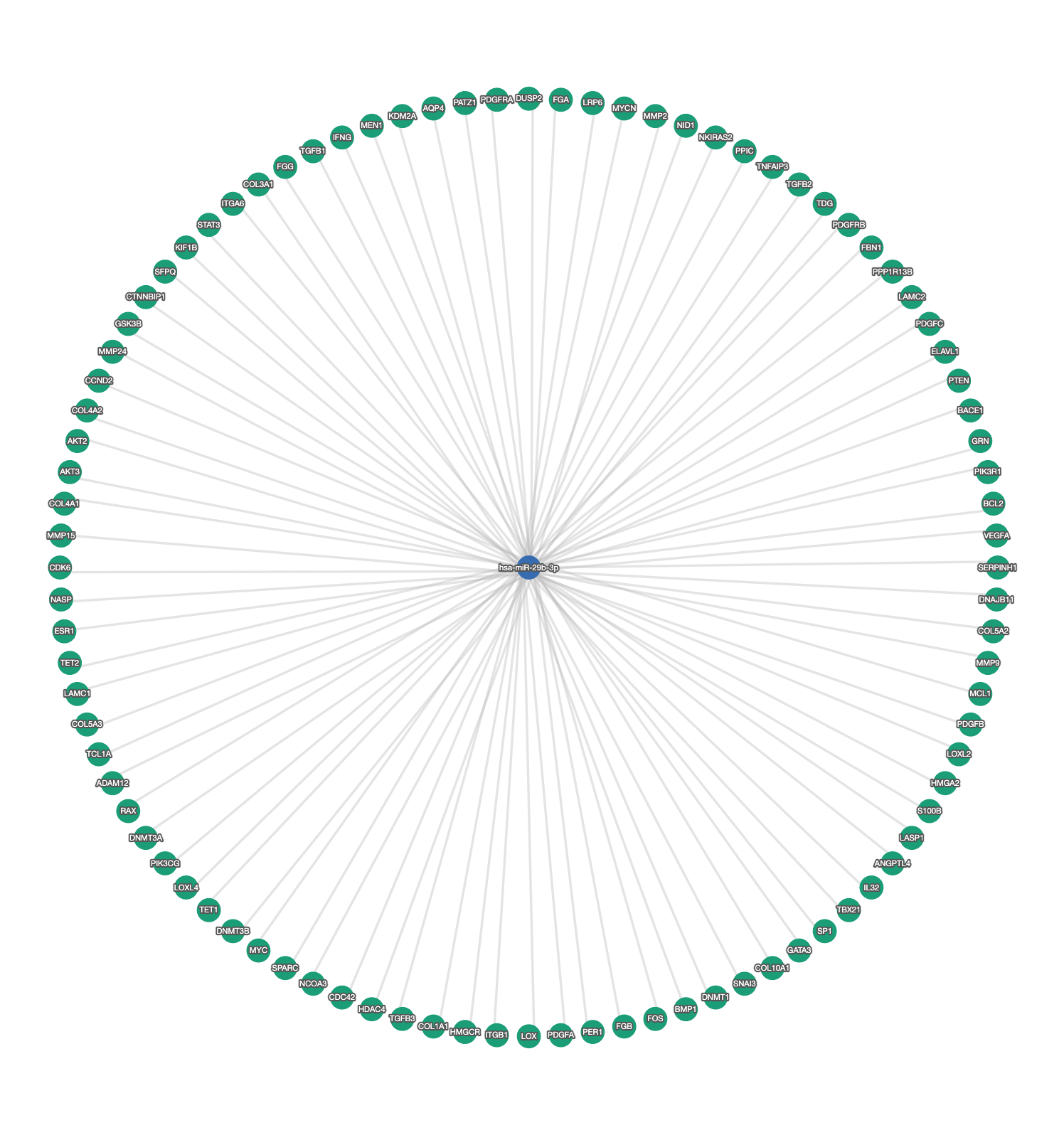


**Figure S3.** Interaction network of miRNA-29b-3p and its validated target genes. Data was retrieved using miRTargetLink 2.0. (https://ccb-compute.cs.uni-saarland.de/mirtargetlink2/).


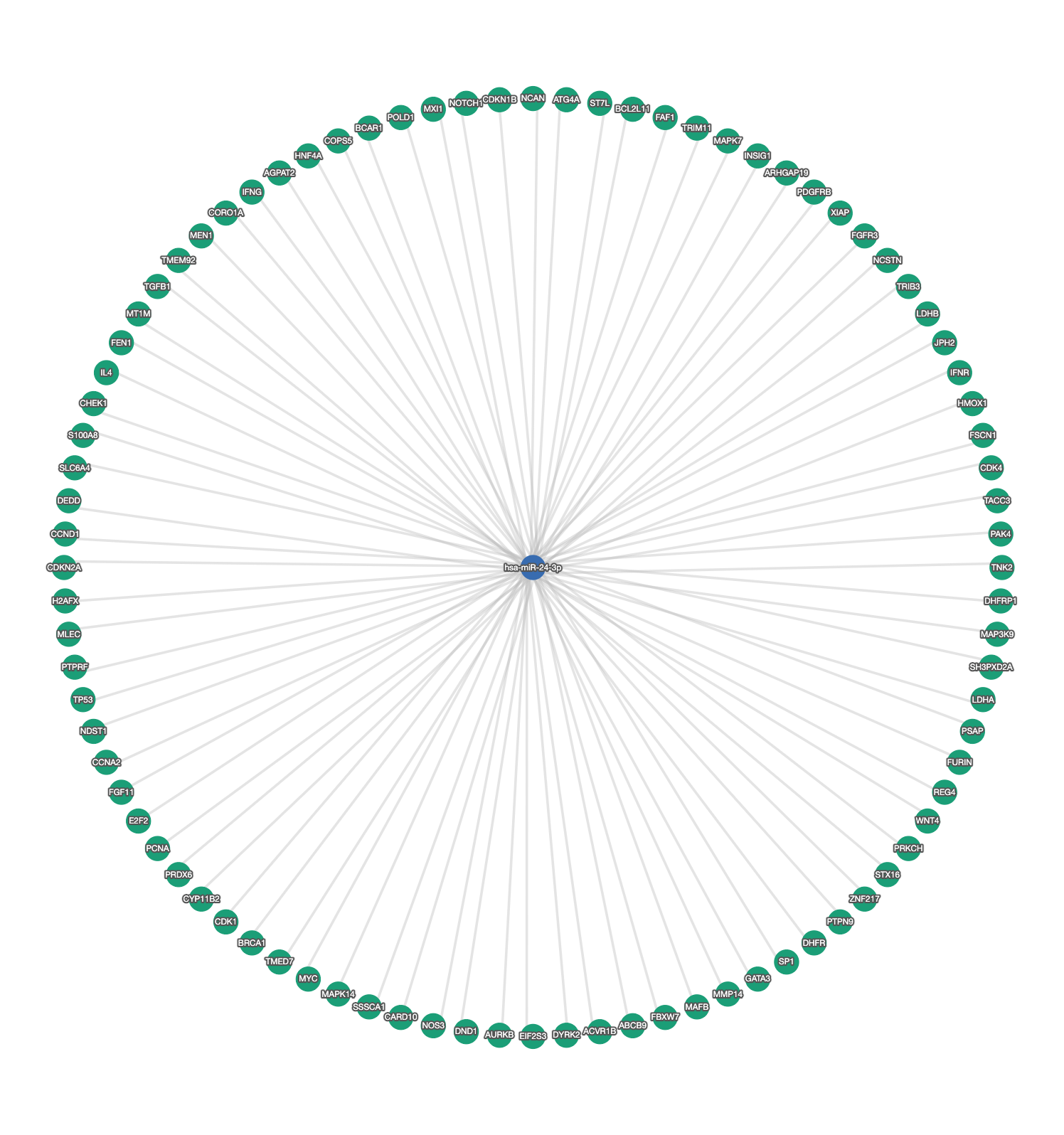


**Figure S4.** Interaction network of miRNA-24-3p and its validated target genes. Data was retrieved using miRTargetLink 2.0. (https://ccb-compute.cs.uni-saarland.de/mirtargetlink2/).


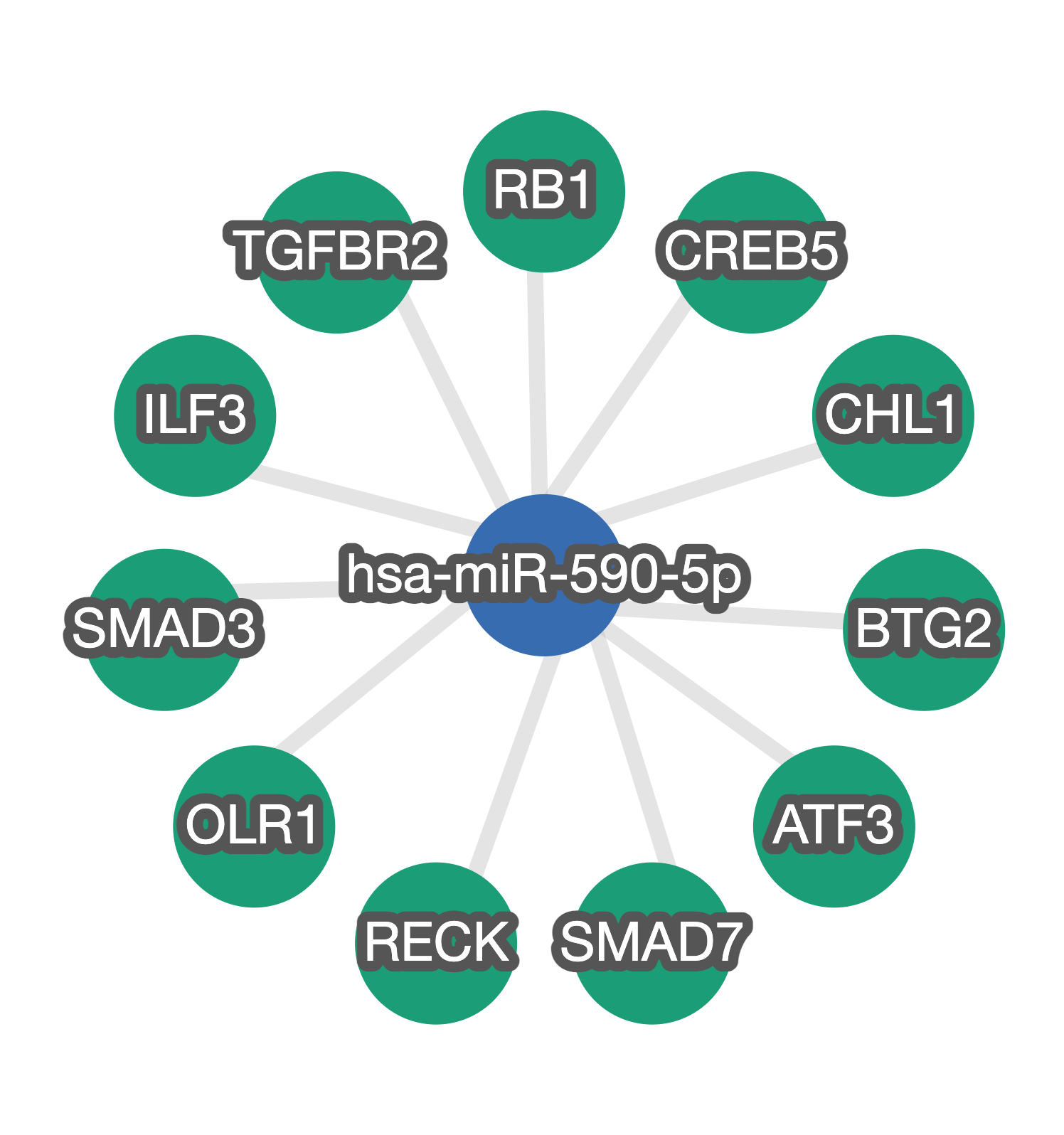


**Figure S5.** Interaction network of miRNA-590-5p and its validated target genes. Data was retrieved using miRTargetLink 2.0. (https://ccb-compute.cs.uni-saarland.de/mirtargetlink2/).
